# Olmesartan‐Associated Enteropathy: An Uncommon Cause of Chronic Diarrhea

**DOI:** 10.1002/ccr3.70706

**Published:** 2025-07-29

**Authors:** Ahmed Sayedahmed, Hisham Sherfi, Mohamed Elobaid, Mohamed Ibrahim, Essam Arabi, Suhaib Ahmed

**Affiliations:** ^1^ Omdurman Islamic University Omdurman Sudan; ^2^ University of Khartoum Khartoum Sudan; ^3^ University of Gezira Al Jazirah Sudan

**Keywords:** chronic diarrhea, drug‐induced enteropathy, Olmesartan, sprue‐like enteropathy

## Abstract

Olmesartan‐associated sprue‐like enteropathy is an uncommon side effect and should be considered as a differential diagnosis in patients who are on Olmesartan and presenting with chronic unexplained diarrhea.

## 1 Introduction

Olmesartan is one of the angiotensin II receptor blockers (ARBs) drugs used for the management of hypertension; it was approved by the FDA in 2002 [1]. Olmesartan‐associated enteropathy is a rare side effect related to Olmesartan, and it is difficult to diagnose due to histological and clinical resemblances to other conditions such as celiac disease [2, 3]. It can cause villous atrophy and malabsorption. This association was first reported by Alberto Rubio‐Tapia et al. [2].

There are other medications reported to cause villous atrophy including azathioprine, colchicine, methotrexate, and neomycin [4, 5, 6]. Villous atrophy is often linked to celiac disease; however, it is unlikely with negative serology (IgA tTGA) and non‐response to the gluten‐free diet [2]. Moreover, other causes of sprue‐like enteropathy should be excluded before considering Olmesartan.

## 2 Case History and Examination

A 74‐year‐old female with a past medical history of hypertension, ischemic heart disease, atrial fibrillation, and congestive cardiac failure presented with watery diarrhea for a week that did not contain blood or mucus. The diarrhea occurred 4–6 times per day. Fever, abdominal pain, and vomiting were denied. She is not a smoker and has not been out of Ireland in the last 6 months. Abdominal examination was unremarkable, and vital signs were stable. The patient was on Olmesartan, Bisoprolol, Clopidogrel, Atorvastatin, and Clexane.

## 3 Methods (Differential Diagnosis, Investigations, and Treatment)

Routine investigations were done, which showed acute kidney injury and hypokalemia (Table 1). Intravenous fluids and potassium were commenced with close monitoring. Infectious diarrhea, celiac disease, and drug‐induced enteropathy were the major differential diagnoses. Furthermore, the general practitioner initially managed the patient as infectious diarrhea with empirical antibiotics, which did not show any improvement. Thereafter, stool tests including *Clostridium difficile* , *Staphylococcus aureus* , Yersinia, Salmonella, Shigella, Campylobacter, *Escherichia coli*, Cryptosporidium, Giardia, *Vibrio cholerae* , Rotavirus, and Norovirus were all negative.

**TABLE 1:** Laboratory results on the day of admission.

Investigation	Value	Reference range
WBC (*10^9^/L)	9.3	4–11
Hemoglobin (g/dL)	12.4	11.5–16.5
CRP (mg/L)	27	0–5
Potassium (mmol/L)	2.8	3.5–5.1
Sodium (mmol/L)	135	136–145
Magnesium (mmol/L)	0.72	0.65–0.98
Urea (mmol/L)	6.8	3.5–7.2
Creatinine (umo/L)	157	49–90

**FIGURE 1:**
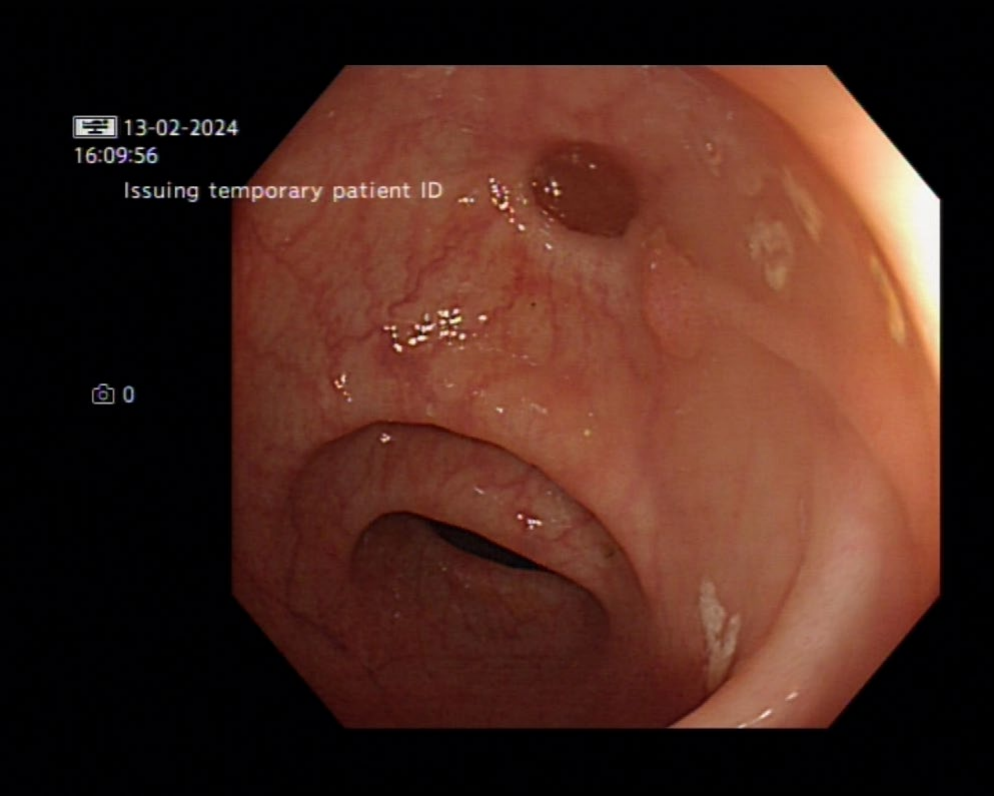
Colonoscopy showing diverticulosis in the sigmoid colon.

**FIGURE 2:**
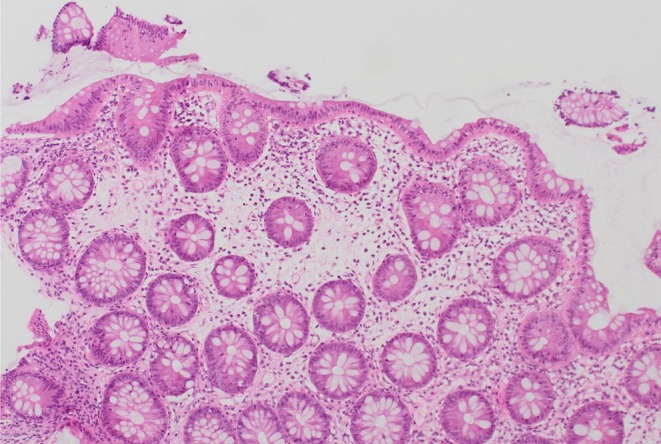
Areas of normal colonic mucosa.

**FIGURE 3:**
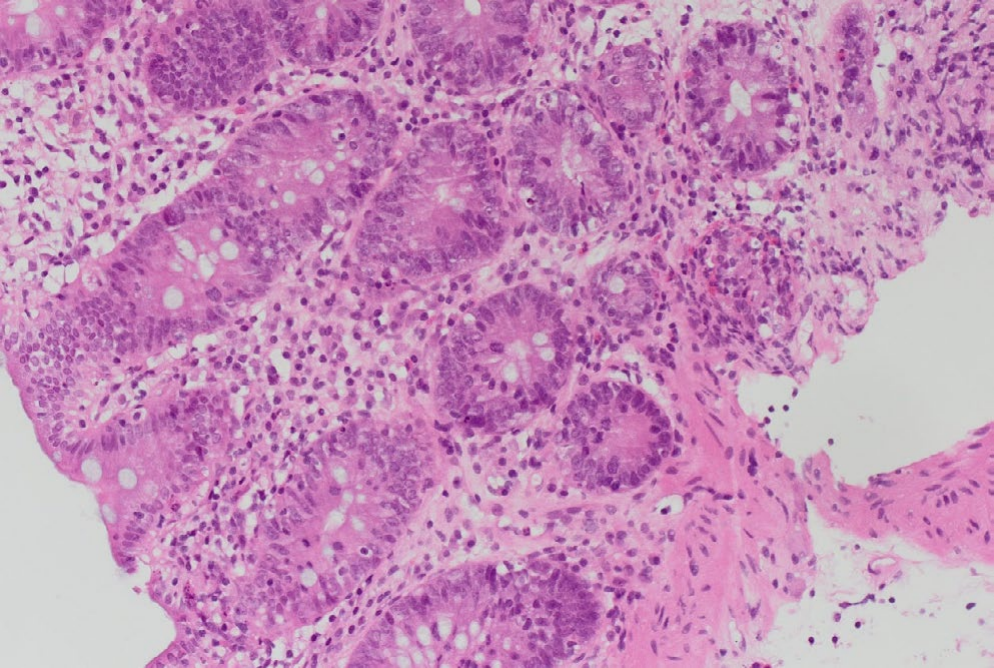
Abnormal right colonic area—inflamed and reactive changes in crypts.

Computed tomography (CT) of the abdomen‐pelvis and abdominal x‐ray revealed no significant abnormal findings. The Esophagogastroduodenoscopy (EGD) revealed fundal polyps, and a colonoscopy was performed subsequently, which only showed diverticulosis in the sigmoid and distal descending colon (Figure 1). In addition, biopsies of the duodenum revealed villous flattening. Multiple biopsies were taken from the colon (Figures 2 and 3). The findings in both the right and left colon revealed crypt inflammation, with infiltration of the lamina propria by polymorphonuclear neutrophils and lymphocytes, and varying degrees of severity, which are more pronounced in the right colon (Figure 3). These features are suggestive of drug‐induced enteropathy. Therefore, Olmesartan‐associated enteropathy was suspected, and Olmesartan was held, which caused gradual improvement of diarrhea. The patient was started on different antihypertensive medication.

## 4 Conclusion and Results (Outcome and Follow‐Up)

A week later, after the cessation of Olmesartan, the patient's condition improved, and the diarrhea completely stopped. On further follow‐up, she reported full resolution of symptoms.

## 5 Discussion

In this study, the patient reported watery diarrhea for a week, which was not associated with blood or mucus. The diarrhea occurred about 5 times daily. The patient denied having fever, abdominal pain, or vomiting. The cessation of Olmesartan was enough to resolve the symptoms.

Chronic diarrhea can be due to different causes such as celiac disease, inflammatory bowel disease, tropical sprue, infections, medication‐induced enteropathy, and other causes. A number of medications can cause enteropathy such as ARBs (e.g., Olmesartan), immunosuppressants, and nonsteroidal anti‐inflammatory drugs (NSAIDs). Therefore, a thorough medication history is important [2, 7]. The absence of antibodies and lack of response to the gluten‐free diet differentiate sprue‐like enteropathy from celiac disease [2]. Crypt apoptosis and villous atrophy may be shown in drug‐induced enteropathy [8].

The most common symptoms are chronic diarrhea and weight loss, followed by nausea, vomiting, and abdominal pain [7, 9]. This can lead to severe dehydration and acute kidney injury.

The exact mechanism underlying Olmesartan‐induced enteropathy is still unclear, but the delay between the commencement of Olmesartan and the onset of symptoms suggests a cell‐mediated immunity [7, 10]. Some studies reported that patients with autoimmune diseases have a higher tendency to develop Olmesartan‐induced enteropathy [11].

The discontinuation of Olmesartan will result in an improvement in the clinical picture. In our case, the patient reported alleviation of symptoms after 1 week of cessation of Olmesartan. In another case report, the clinical picture started to improve 3 days after Olmesartan was stopped [10]. Furthermore, restarting the Olmesartan is not recommended because this can lead to recurrence of symptoms [12, 13].

## Author Contributions

**Ahmed Sayedahmed:** conceptualization, data curation, writing – original draft. **Hisham Sherfi:** supervision, writing – review and editing. **Mohamed Elobaid:** writing – original draft, writing – review and editing. **Mohamed Ibrahim:** data curation, investigation. **Essam Arabi:** writing – original draft, writing – review and editing. **Suhaib Ahmed:** resources, software, writing – review and editing.

## Ethics Statement

Patient confidentiality was maintained in this report, and all ethical considerations were done in accordance with the Declaration of Helsinki.

## Consent

A written informed consent was obtained from the patient prior to the writing and submission of this case report.

## Conflicts of Interest

The authors declare no conflicts of interest.

## Data Availability

The data that support the findings of this study are available from the corresponding author upon reasonable request.
